# Antiviral Potential of Sea Urchin Aminated Spinochromes against Herpes Simplex Virus Type 1

**DOI:** 10.3390/md18110550

**Published:** 2020-11-05

**Authors:** Natalia P. Mishchenko, Natalia V. Krylova, Olga V. Iunikhina, Elena A. Vasileva, Galina N. Likhatskaya, Evgeny A. Pislyagin, Darya V. Tarbeeva, Pavel S. Dmitrenok, Sergey A. Fedoreyev

**Affiliations:** 1 G.B. Elyakov Pacific Institute of Bioorganic Chemistry, Far-Eastern Branch of the Russian Academy of Sciences, 690022 Vladivostok, Russia; vasilieva_el_an@mail.ru (E.A.V.); galinlik@piboc.dvo.ru (G.N.L.); pislyagin@hotmail.com (E.A.P.); tarbeeva1988@mail.ru (D.V.T.); paveldmitrenok@mail.ru (P.S.D.); fedoreev-s@mail.ru (S.A.F.); 2 G.P. Somov Institute of Epidemiology and Microbiology, Far-Eastern Branch of the Russian Academy of Sciences, 690087 Vladivostok, Russia; krylovanatalya@gmail.com (N.V.K.); olga_iun@inbox.ru (O.V.I.)

**Keywords:** echinochrome A, echinamine A, echinamine B, herpes simplex virus type 1, Vero cells, glycoprotein gD, molecular docking

## Abstract

Herpes simplex virus type 1 (HSV-1) is one of the most prevalent pathogens worldwide requiring the search for new candidates for the creation of antiherpetic drugs. The ability of sea urchin spinochromes—echinochrome A (EchA) and its aminated analogues, echinamines A (EamA) and B (EamB)—to inhibit different stages of HSV-1 infection in Vero cells and to reduce the virus-induced production of reactive oxygen species (ROS) was studied. We found that spinochromes exhibited maximum antiviral activity when HSV-1 was pretreated with these compounds, which indicated the direct effect of spinochromes on HSV-1 particles. EamB and EamA both showed the highest virucidal activity by inhibiting the HSV-1 plaque formation, with a selectivity index (SI) of 80.6 and 50.3, respectively, and a reduction in HSV-1 attachment to cells (SI of 8.5 and 5.8, respectively). EamA and EamB considerably suppressed the early induction of ROS due to the virus infection. The ability of the tested compounds to directly bind to the surface glycoprotein, gD, of HSV-1 was established in silico. The dock score of EchA, EamA, and EamB was −4.75, −5.09, and −5.19 kcal/mol, respectively, which correlated with the SI of the virucidal action of these compounds and explained their ability to suppress the attachment and penetration of the virus into the cells.

## 1. Introduction

According to the World Health Organization, herpes simplex virus (HSV) infections are the second most common viral disease in humans after influenza [1]. HSV type 1 (HSV-1) is a neurotropic virus that can persist in human sensory neurons for life, replicate in epithelial cells during primary infection and reactivation, and cause diseases with a variety of clinical manifestations, from labial herpes to meningitis and encephalitis [2]. Recently, the number of reports on the resistance of HSV-1 against many drugs based on nucleoside analogues, which are acyclovir and its derivatives, has increased [3,4,5]. Therefore, the search for new antiviral drugs, specifically anti-HSV drugs, remains an important problem.

Many of the cytopathic effects observed during HSV-1 infection are not only due to viral replication but also to oxidative stress caused by the virus, leading to cell damage [6,7,8]. Therefore, using exogenous antioxidants capable of counteracting the destructive effect of virus-induced oxidative stress on cells is considered a promising strategy [9]. Currently, many laboratories around the world are actively studying antioxidants with different chemical structures as potential therapeutic agents for herpes infection treatment. The largest number of studies on the antiherpetic activity of natural antioxidants is associated with the study of polyphenolic compounds from terrestrial and marine plants [10,11]. In this regard, a class of marine natural polyphenolic compounds found in representatives of Echinodermata, such as sea urchins, sea stars, and ophiurs, is promising [12]. This class is called spinochromes and has been known for over 100 years; nevertheless, extensive investigations of the biological activities of these compounds began only in the last decade [13,14].

Recently, our group discovered the antiviral properties of the most abundant and well-known spinochrome, echinochrome A (EchA), as well as an antioxidant composition consisting of EchA in combination with ascorbic acid and α-tocopherol. It was found that EchA and the composition effectively inhibit the cytopathogenic effect of HSV-1 and tick-borne encephalitis virus [15]. EchA and the antioxidant composition have shown a considerable protective effect on the mouse model of genital HSV type 2 (HSV-2) infection. Both intraperitoneal and oral administration of EchA and the composition significantly improved the survival rate and reduced the vaginal virus load of HSV-2-infected mice [16]. The potential affinity of sea urchin pigments to bind the main protease (Mpro) and the spike protein (S) of SARS-CoV-2 was evaluated in silico [17,18]. EchA showed high affinity to both targets, which indicated its potential as an antiviral drug against SARS-CoV-2 and encouraged further in vitro and in vivo analysis to expand its therapeutic uses.

The first spinochromes containing a primary amino group, echinamines A (EamA) and B (EamB), which are aminated derivatives of EchA, were isolated in 2005 from the flat sea urchin *Scaphechinus mirabilis* [19]. Nowadays, amino derivative of spinochrome E—spinamine E [20] has also been isolated from various species of sea urchins, and the new aminated quinonoid pigment acetylaminotrihydroxynaphthoquinone [21] was detected in the sea urchin *Strongylocentrotus nudus* using UPLC-DAD-MS (ultra performance liquid chromatography-diode array detector-mass spectrometry). The natural origin of these spinochromes is beyond doubt; they were found in both the regular sea urchins *Strongylocentrotus nudus*, *S. pallidus*, and *S. polyacanthus* and the irregular sea urchins *Echinarachnius parma* and *Scaphechinus mirabilis*, which inhabit the southern and northern seas at different depths up to 200 m [20,21,22,23]. The simultaneous presence of three aminated spinochromes—EamA, EamB, and spinamine E—was found in the commercial sea urchin *Evechinus chloroticus* from New Zealand, also known as kina [23].

A number of investigations have reported that spinochromes are strong antioxidants that can block a number of free radical reactions, inhibit lipid peroxidation, and reduce and chelate metal ions [21,24,25,26,27]. Recently, using 2,2-diphenyl-1-picrylhydrazyl radical (DPPH) and lipid autooxidation assays, it has been shown that aminated spinochromes EamA and EamB and spinamine E are more potent antioxidants than their hydroxylated analogues [20].

Thus, the aim of this study was to evaluate the anti-HSV-1 activity of EamA and EamB in vitro and in silico as well as their effect on the HSV-1-induced production of reactive oxygen species (ROS) in Vero cells, in comparison with EchA.

## 2. Results and Discussion

### 2.1. Physico-Chemical Properties of Aminated Spinochromes

**Figure 1:**
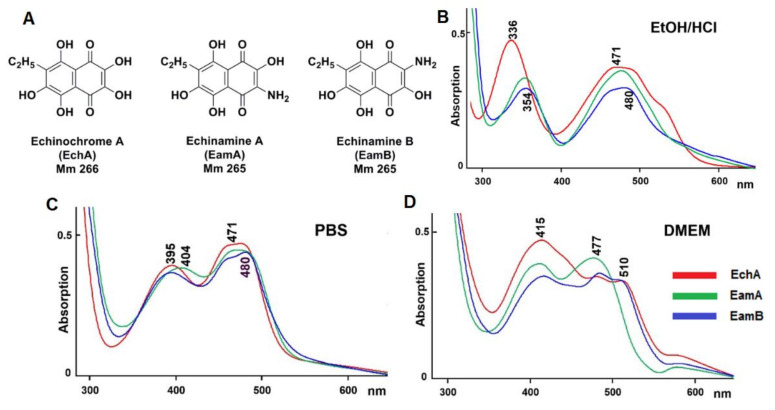
(**A**) Structural formulae of the studied spinochromes. Absorption spectra of the studied spinochromes (0.02 mg/mL) in (**B**) acidified ethanol (pH 1.0), (**C**) phosphate buffered saline (PBS); 0.01 M, pH 7.2, and (**D**) Dulbecco’s Modified Eagle’s Medium (DMEM) (pH 7.2). Compound spectra were recorded in comparison to the corresponding solvent.

Spinochromes of the sea urchin *Echinarachnius parma* were isolated using a previously described standard procedure [20]: shells were treated with ethanol containing 10% sulfuric acid, and the sum of the pigments was extracted with chloroform and ethyl acetate and then fractionated using repeated column chromatography on Toyopearl HW-40 gel (TOYO SODA, Tokyo, Japan). As a result, EchA, EamA, and EamB were isolated (Figure 1A). According to HPLC-MS (high performance liquid chromatography mass spectrometry) data, *E. parma* extracts also contained spinochromes D and E and three binaphthoquinones [22], which were not used in this study.

The content of aminated spinochromes in *E. parma* was not higher than 3% of the total amount of quinonoid pigments, which was not enough for biological activity investigations. Recently, several simple and effective synthesis schemes of EamA and EamB have been described [28,29,30,31,32]. However, the most promising method developed by us for obtaining EamA and EamB is the amination of EchA with aqueous ammonia [29]. This reaction led to the formation of a mixture of EamA and EamB in nearly quantitative overall yield, which was successfully separated by column chromatography on a Sephadex LH-20. The HPLC-MS and NMR characteristics of the obtained EamA and EamB coincided with the data of natural echinamines (Supplementary Materials, page 3, Figure S1).

Earlier, we described two specific differences of the aminated spinochromes from their hydroxylated analogues. They have one unit less molecular weight compared to the corresponding hydroxylated spinochrome, and the quinoid electron transfer band in the absorption spectrum of the aminated spinochromes is usually 10–15 nm bathochromically shifted compared with that of their hydroxylated analogues [20]. As seen from in Figure 1B, this is true for both EamA and EamB.

It is known that small-molecule drug substances are ionisable under physiological conditions, forming a number of protomers [33]. Under physiological conditions, for example, in phosphate buffer PBS pH 7.2, all studied spinochromes become ionisable, reflecting a shift in the quinoid absorption band to about 400 nm (Figure 1C). The possible protomeric structures of each spinochrome in the aqueous phase with physiological pH were calculated using MOE 2019.01 software (Figure S2). Under these conditions, echinochrome A forms the largest number of protomers, in which the quinoid fragment of the molecule is presented in the form of triketone, and the negative charge is delocalised in the benzene fragment. The smallest number of protomers was obtained for EamB, one of which takes 77% of the possible structures. Knowledge of the structures of protomers is important for predicting their properties since they determine the binding with biological molecules [34,35]. The absorption spectra of spinochromes in Dulbecco’s Modified Eagle’s Medium (DMEM) containing glucose, vitamins, salts, l-glutamine, and other amino acids, differed significantly (Figure 1D), possibly due to the presence of different protomeric forms of these compounds in solution, which define the binding with the components of the DMEM.

### 2.2. Cytotoxicity and Anti-HSV-1 Activity of the Tested Compounds

The cytotoxicity of the spinochromes and acyclovir (ACV) as a positive control against Vero cells was evaluated before testing their antiviral activity. Cells were treated with concentrations of spinochromes from 1 to 200 μg/mL and of ACV from 1 to 1000 μg/mL for 72 h, and cell viability was assessed using the MTT (methylthiazolyltetrazolium bromide) assay. There was no significant difference (*p* > 0.05) between the 50% cytotoxic concentrations (CC_50_) of the studied spinochromes; their mean CC_50_ values were ~140 μg/mL, while the CC_50_ of ACV was above 1000 μg/mL (Table 1). Therefore, the antiviral activity of the tested compounds against HSV-1 was determined at concentrations below 100 μg/mL.

The inhibitory effect of the tested compounds on different stages of HSV-1 infection was studied by a plaque reduction assay. We used the following treatment schemes [36]: pretreatment of the virus (compounds were added directly to the virus suspension); pretreatment of the cells (cells were treated with compounds for 1 h before infection); attachment (cells were co-treated with the virus and compounds at 4 °C); penetration (cells were infected with the virus at 4 °C and then treated with the compounds at 37 °C); and simultaneous and post-infection treatment (cells were treated with the compounds at the same time as infection or 1 h after infection). The antiviral effect of the tested compounds was compared to that of the untreated virus after 72 h of incubation, and the obtained results were used for calculations of the concentration yielding a 50% reduction in plaque formation (IC_50_) and the selectivity index (SI) as the ratio of CC_50_ to IC_50_ for each of the compounds.

The pretreatment of HSV-1 (100 plaque-forming units (PFU)/mL) with different concentrations (0.2 to 25 μg/mL) of the tested spinochromes and ACV showed that the spinochromes significantly inhibited HSV-1-induced plaque formation, with IC_50_ values of less than 5 μg/mL (Table 1, Figure S3). Among the three tested compounds, EamB was found to have the highest SI value (80.6) against HSV-1. In comparison, ACV did not show antiviral activity, even when tested up to a concentration of 100 μg/mL. These results indicate the direct effect of spinochromes on HSV-1 particles, with the virucidal activity of EamA and EamB being significantly higher (*p* ≤ 0.05) than that of EchA.

The treatment of Vero cells with the tested compounds before infection (pretreatment of cells) had no effect on HSV-1 plaque formation. Post-infection treatment of cells with spinochromes was also ineffective in contrast to ACV (SI of ~1.5 vs. 10,000), which is known to block viral replication [37]. Next, the tested compounds and HSV-1 were added to Vero cells simultaneously to investigate their influence on the early stages of viral infection. In this assay, spinochromes displayed moderate inhibitory activity against HSV-1 (IC_50_ > 30 μg/mL; SI of ~4.3) (Table 1).

Thus, the results of HSV-1 pretreatment with spinochromes and the results of simultaneous treatment of cells with HSV-1 and spinochromes revealed that these compounds affect the very early stages of the HSV-1 life cycle, which are the attachment and penetration stages.

The attachment and penetration stages were analysed by shifting the temperature during the infection. It is known that for many enveloped viruses, including HSV-1, a temperature of 4 °C allows for virus attachment to host cells, but not cell penetration, whereas a temperature of 37 °C facilitates virus penetration into the cells [38,39,40]. In the attachment assay, during which Vero cells were co-treated with HSV-1 and tested compounds at 4 °C, EamA and EchA displayed a moderate inhibitory effect on the binding of the virus to cells (Table 1). At the same time, EamB significantly reduced (*p* ≤ 0.05) the HSV-1 attachment to cells (SI = 8.5) compared to EchA (SI = 4.3). For the penetration assay, cells were infected with HSV-1 at 4 °C and then treated with test compounds at 37 °C to facilitate virus entry into the cells. The results showed that the effect of spinochromes on viral entry was not significant (SI of ~2.5). ACV did not affect the attachment and penetration stages of HSV-1 infection when tested up to a concentration of 100 μg/mL.

Thus, it was shown that the tested spinochromes (EamA, EamB, and EchA) exert significant anti-HSV-1 activity, mainly due to their direct virucidal properties, but they also inhibit the attachment of the virus to cells and, to a lesser extent, virus penetration into cells.

### 2.3. Effect of Spinochromes on HSV-1-Induced Intracellular ROS Production

**Figure 2:**
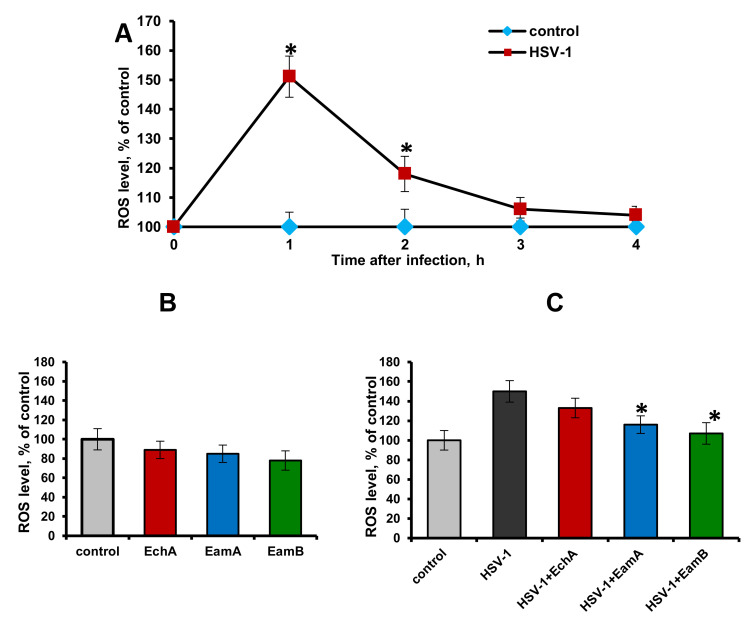
Effects of HSV-1 and spinochromes on reactive oxygen species (ROS) levels in Vero cells. The DCF-DA (2′,7′-dichlorofluorescein diacetate) assay was used to measure the cellular ROS. (**A**) Dynamics of HSV-1-induced intracellular ROS levels compared to uninfected cells (control). Data are presented as the mean ± SD of three independent experiments. * *p* ≤ 0.05 compared with the control. (**B**) Effects of the studied spinochromes (5 µg/mL) on the ROS levels in uninfected cells. (**C**) Effects of HSV-1 (100 PFU/mL) on ROS levels after incubation for 1 h with spinochromes (5 µg/mL). The results include data from three experiments (mean ± SD). * *p* ≤ 0.05 compared with HSV-1-infected cells.

To determine whether HSV-1 infection induced ROS production, Vero cells were infected with HSV-1 at 100 PFU/mL. At different time points after infection (1, 2, 3, and 4 h), the cells were loaded with the fluorogenic marker (DCF-DA (2′,7′-dichlorofluorescein diacetate)), and ROS production was assessed by changes in the mean fluorescence intensity in the control (uninfected cells) versus the infected cells. The results reported in Figure 2A show that HSV-1 infection of Vero cells induced a significant increase in ROS production at 1 and 2 h post-infection (*p* ≤ 0.05), but at 3 and 4 h post-infection the levels of ROS production in the infected cells did not differ from those in the control. These results indicated that HSV-1 was able to induce a high level of ROS at a very early stage of infection, which has also been confirmed by other authors [8,41].

The next task was to find out whether the spinochromes could affect the HSV-1-induced ROS production in Vero cells. Since the tested compounds exhibited significant anti-HSV-1 activity, mainly due to direct inactivation of viral particles, as noted above, to study their effect on virus-induced ROS production, HSV-1 was pre-incubated with these compounds. In these experiments, spinochromes were used at a concentration of 5 μg/mL, which is approximately equal to the IC_50_ concentration calculated for them in the virucidal assay. At first, it was found that the tested compounds themselves, when added to Vero cells for 2 h, did not cause a significant decrease in ROS production compared to the cell control (*p* > 0.05) (Figure 2B). At the same time, pretreatment of HSV-1 (100 PFU/mL) with EamA and EamB followed by the 1 h exposure of these mixtures on Vero cells significantly inhibited the early induction of ROS formation by the virus compared with untreated HSV-1-infected cells (*p* ≤ 0.05) (Figure 2C).

### 2.4. Molecular Docking

HSV-1 surface glycoproteins are involved in the processes of adhesion and fusion of the virus with the cell membrane. The HSV-1 glycoprotein gD is involved in the interaction of the virus with the cellular receptors nectin-1, HSEA/M, and 3-O-sulphated heparan sulphate (3-OS HS) [42,43]. The interaction of the gD protein with the receptors triggers the adhesion of the virus to the cell and fusion with the cell membrane. This protein can be used as a target protein to search for compounds that inhibit the initial stages of HSV-1 infection. It was shown with molecular docking that flavonoid myricetin can block HSV infection by directly interacting with the viral gD protein and interfering with virus adsorption [44].

In this work, the interaction of spinochromes with the HSV-1 surface glycoprotein, gD, was studied using molecular docking methods, and it was shown that the studied compounds can directly bind to gD, competing for binding sites of this protein with cellular receptors (nectin-1 and 3-OS HS), thereby preventing the adsorption of the virus to cells.

**Figure 3:**
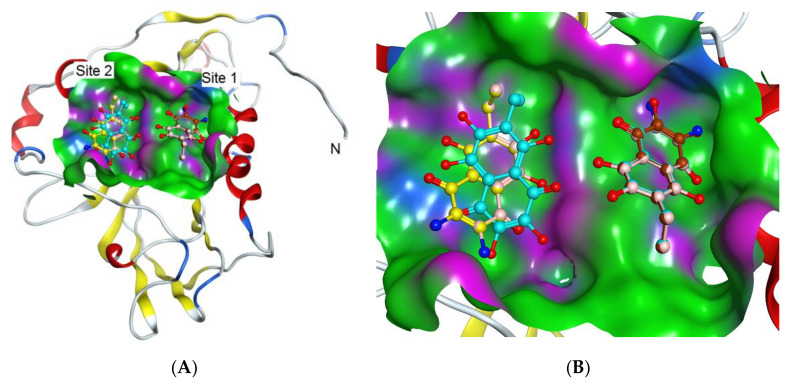
Molecular docking of spinochromes with gD. (**A**) Subsites 1 and 2 of binding of spinochromes to gD. Spinochrome structures are shown in colour as balls and sticks. The gD molecule is shown as a ribbon. (**B**) Molecular surface of gD in the binding sites of the spinochromes. Site 1: EchA (turquoise), EamA (beige), EamB (brown); Site 2: EchA (turquoise), EamA (beige), EamB (yellow). The colour shows the molecular surface of gD: H-bonding (pink), hydrophobic (green), and mild polar (blue).

**Figure 4:**
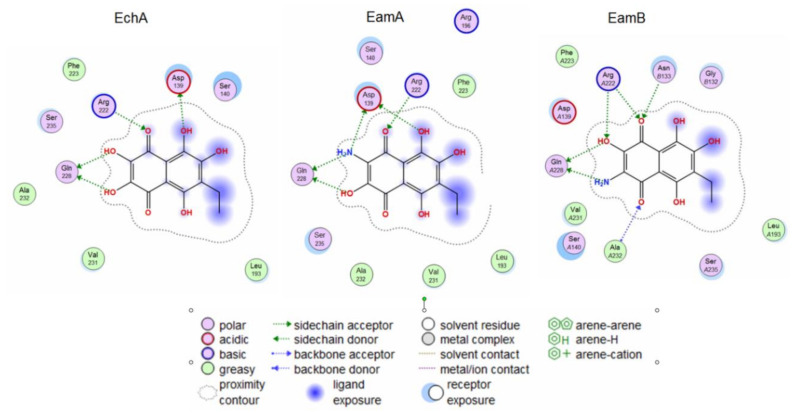
2D diagrams of EchA, EamA, and EamB contacts with the HSV-1 glycoprotein gD. Abbreviations of aminoacids: alanine (Ala); arginine (Arg); aspartic acid (Asp); asparagine (Asn); glutamine (Gln); glycine (Gly); leucine (Leu); phenylalanine (Phe); serine (Ser); valine (Val).

The search for binding sites for the gD glycoprotein showed that one of the sites overlaps with the site for gD binding to the nectin-1 cellular receptor (Figure S4). Molecular docking of spinochromes into this site showed that spinochromes have two subsites on gD (Figure 3). Analysis of the contacts of the spinochromes with gD showed that the compounds under study form hydrogen bonds with amino acid residues of gD, including the arginine (Arg) 222 residue, which is important for binding to the nectin-1 receptor (Figure 4).

Apparently, the interaction of spinochromes with Arg 222 inhibits the binding of gD to the cellular receptor, nectin-1. The dock score of EchA, EamA, and EamB in Subsite 1 with gD was −4.75, −5.09, and −5.19 kcal/mol, respectively, and correlated with the SI values obtained in the virucidal and attachment analysis (Table 1).

**Figure 5:**
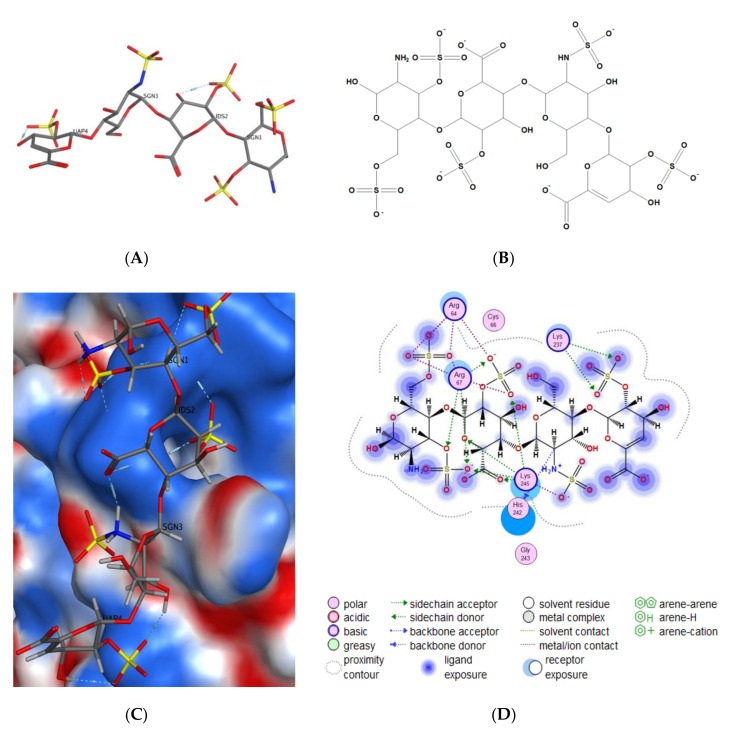
Molecular docking of the 3-*O*-sulphated heparan sulphate (3-OS HS) tetrasaccharide and the herpes simplex virus type 1 (HSV-1) gD membrane glycoprotein. (**A**) 3D structure and (**B**) 2D structure of 3-OS HS obtained using the MOE program. (**C**) The putative binding site of 3-OS HS and gD HSV-1. (**D**) 2D diagram of the contacts of 3-OS HS with gD.

**Figure 6:**
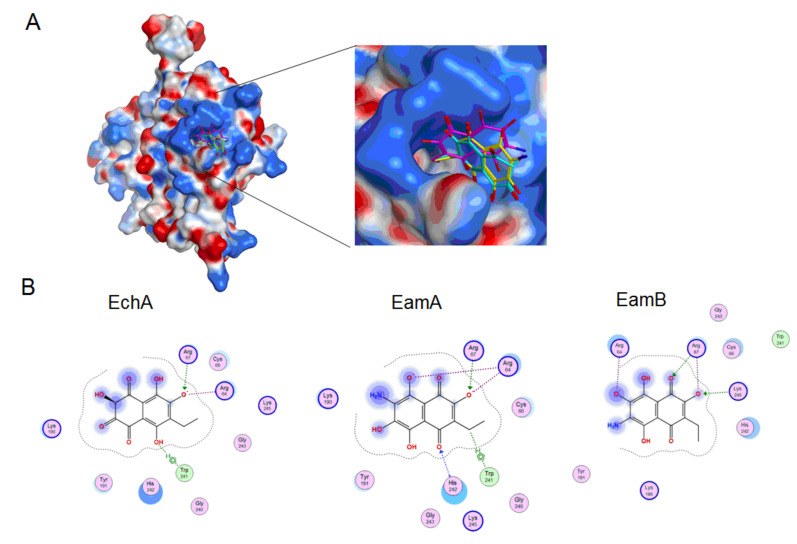
(**A**) Molecular docking of spinochromes into a potential binding site of the 3-OS HS tetrasaccharide and the HSV-1 gD membrane glycoprotein. The electrostatic potential of the gD molecular surface was calculated using the MOE 2019.01 program. The spinochrome structures are shown as sticks. (**B**) 2D diagram of contacts of EchA, EamA, and EamB protomers with gD.

The interaction of gD and 3-OS HS is a key step for triggering the fusion of the virus and cells [43]. Calculation of the electrostatic potential of the molecular surface of the gD protein showed that there are positively charged sites on the surface of the viral protein that can potentially interact with 3-OS HS. The interactions of the 3-OS HS tetrasaccharide and the spinochromes with the glycoprotein, gD, at a positively charged site were tested. It was shown by molecular docking that the 3-OS HS tetrasaccharide and the spinochromes compete for interaction with Arg 67 and Arg 64 residues of the gD protein (Figure 5 and Figure 6). The in silico results allowed for a better understanding and clarification of the mechanism underlying the in vitro anti-HSV-1 action of the spinochromes. The ability of the tested compounds to directly bind to the gD surface of the HSV-1 virus, to compete with the cellular receptors for binding sites on this glycoprotein, and to suppress the attachment and penetration of the virus into the cells was established. Blocking the early stages of HSV-1 infection may be an attractive therapeutic strategy, the advantage of which is that it can prevent the spread of the virus without killing virus-infected cells, as occurs with acyclovir.

## 3. Materials and Methods

### 3.1. Reagents

DMEM (Biolot, St. Petersburg, Russia), fetal bovine serum (FBS, Biolot, Saint Petersburg, Russia), gentamycin (Dalkhimprom, Khabarovsk, Russia), Acyclovir^®^ (ACV; freeze-dried powder for injections; GlaxoSmithKline Pharmaceuticals S.A., Poznan, Poland), dimethylsulfoxide (DMSO; Sigma, Saint-Louis, MO, USA), methylthiazolyltetrazolium bromide (MTT; Sigma, Saint-Louis, MO, USA), 2′,7′-dichlorofluorescein diacetate (DCF-DA; Sigma, Saint-Louis, MO, USA), carboxymethyl cellulose (CMC; MP Biomedicals, Inc., Aurora, OH, USA), crystal violet (Sigma, Saint Louis, MO, USA), and phosphate buffered saline (PBS, Sigma, Saint-Louis, MO, USA) were used.

### 3.2. Viruses and Cell Cultures

HSV-1 strain L2 was obtained from N.F. Gamaleya Federal Research Centre for Epidemiology and Microbiology, Moscow, Russia. HSV-1 was grown in African green monkey kidney (Vero) cells using DMEM supplemented with 10% FBS and 100 U/mL of gentamycin at 37 °C in a CO_2_ incubator. In the maintenance medium, the FBS concentration was decreased to 1%.

### 3.3. Spinochromes Isolation

The sea urchin *Echinarachnius parma* (Lamarck, 1816) was collected by dredging during the 47th (July 2015) scientific cruise of R/V Academic Oparin, near Iturup Island (45°38′9″ N, 148°22′6″ E; depth of 54 m). EchA was isolated from the sea urchin *E. parma* according to previous work [19]. The purity of EchA was >99% according to the HPLC-MS data (Shimadzu LCMS-2020, Kyoto, Japan).

For EamA and EamB synthesis, 20 mg of echinochrome A was dissolved in 10 mL of ethanol. Then, 5 mL of 25% aqueous ammonia was added, and the mixture was stirred at room temperature. The reaction time was complete in 5 min and acidified to a pH of 1 with 12% hydrochloric acid. The reaction products were extracted with ethyl acetate, the solution was dried with sodium sulphate, and the solvent was removed in vacuo. The residue was chromatographed on a column (1.1 × 40 cm) with Sephadex LH-20 (GE Healthcare Bio-Sciences AB, Uppsala, Sweden) using CHCl_3_–MeOH (8:1) as the eluent. As a result, 8 mg of echinamine A and 8 mg of echinamine B were obtained. Each compound was fully characterised by NMR and HPLC-DAD-MS data in comparison with the authentic samples isolated from *Scaphechinus mirabilis* ([19]; see Supplementary Materials). According to the HPLC-MS data, the purity of echinamines A and B was more than 98% (Shimadzu LCMS-2020, Kyoto, Japan).

The spinochromes were dissolved in DMSO (Sigma, Saint-Louis, MO, USA) at a concentration 10 mg/mL and stored at −20 °C. For cytotoxicity and anti-HSV-1 activity determination, the stock solutions were diluted with DMEM so that the final concentration of DMSO was 0.5%.

For spectrophotometric studies, 3 mL of either EtOH/HCl (pH 2.0), PBS buffer (pH 7.2), or DMEM (pH 7.2) was first placed in the UV cuvette (10 mm), and then a 20 μL aliquot of the 5 mg/mL methanolic solution of the spinochromes was added. The absorbance was recorded in comparison to the corresponding solvent using a UV 1800 spectrophotometer (Shimadzu USA Manufacturing Inc. Canby, OR, USA).

### 3.4. Cytotoxicity of the Tested Compounds

The cytotoxicity evaluation of the studied compounds was performed using the MTT assay, as previously described [45]. In brief, confluent Vero cells in 96-well microplates were incubated with two-fold serial dilutions of the tested compounds (1–1000 μg/mL) at 37 °C for 72 h (5% CO_2_). Untreated cells were used as controls. Then MTT solution (5 mg/mL) was added, and the cells were incubated at 37 °C for 2 h. After dissolution of formazan crystals, optical densities were read at 540 nm (Labsystems Multiskan RC, Vantaa, Finland). The 50% cytotoxic concentrations (CC_50_) of the tested compounds able to reduce cell viability by 50% were calculated using regression analysis-generated data from three independent experiments [46].

### 3.5. Anti-HSV-1 Activity of the Tested Compounds

The inhibitory effects of the tested compounds on HSV-1 replication cycle stages in Vero cells were evaluated by the plaque reduction assay [47,48]. Vero cell monolayers grown in 24-well plates (1 × 10^5^ cells/well) were infected with 100 PFU/mL of HSV-1. The tested compounds and Acyclovir^®^ were used at concentrations from 0.1 to 100 μg/mL.

- *Virucidal assay* (the pretreatment of the HSV-1 with compounds). HSV-1 suspension was pre-incubated with an equal volume of DMEM or various concentrations of tested compounds for 1 h at 37 °C, then the mixture was used to infect cellular monolayers. After viral adsorption for 1 h at 37 °C, the plates were washed, covered with the maintenance medium (DMEM) containing 1% CMC for 72 h at 37 °C (5% CO_2_) until plaques formed.
- *Time-of-addition assay*. The tested compounds, at various concentrations, were added to cells at 1 h before viral infection (pretreatment of cells), at the same time with infection (simultaneous treatment) or 1 h after infection (post-treatment). To study a preventive effect, the cells were pretreated with compounds for 1 h, then infected with HSV-1 for 1 h after removal of compounds by washing and overlaid with DMEM with 1% CMC. To study the effect on virus adsorption, cells were treated with compounds and simultaneously infected with HSV-1, then overlaid with DMEM with 1% CMC after removal of the compounds and unbound virus by washing at 1 h after adsorption. To study the effect on the early stage of virus replication, the cells were infected with the HSV-1 for 1 h, and then overlaid with DMEM with 1% CMC containing different concentrations of the studied compounds after removal of virus by washing. Within all procedures, the cells were incubated for 72 h at 37 °C (5% CO_2_) until plaques formed.

The attachment and penetration assays were performed as described previously [48,49].

- *The attachment assay*. Pre-chilled at 4 °C for 1 h Vero cells were infected with the virus (100 PFU/mL), and incubated for 3 h at 4 °C with different concentrations of the studied compounds. Then, the compounds and unbound viruses were washed away with cold PBS. The cells were supplied with DMEM containing 1% CMC and incubated for 72 h at 37 °C (5% CO_2_) until plaques formed.
- *The penetration assay*. Pre-chilled at 4 °C for 1 h Vero cells were infected with the virus (100 PFU/mL), and incubated for 3 h at 4 °C. The unbound viruses were removed with cold PBS, the cells were treated with medium containing different concentrations of the compounds, and then incubated for 1 h at 37 °C. Viruses that did not enter cells were inactivated with citrate buffer (pH 3.0). Then, the cells were washed with PBS, supplied with DMEM containing 1% CMC, and incubated for 72 h at 37 °C (5% CO_2_) until plaques formed.

In all assays, after 72 h of incubation, the cells were fixed with cold ethanol for 20 min, stained with solution of 0.5% crystal violet in 20% ethanol, and the viral plaques were then counted. The plaque formation inhibition rate was calculated according to the following formula [36]: plaque inhibition (%) = 100 − [(P_T_/P_C_) × 100], where P_T_ and P_C_ refer to the number of plaques in the compound-treated cells and the virus-infected cells, respectively. The IC_50_ of each compound was determined as the concentration that inhibited 50% of viral plaque formation, compared to virus-infected cells, and was calculated using a regression analysis of the dose–response curve [46]. The SI was calculated as the ratio of CC_50_ to IC_50_ for each compound.

### 3.6. Measurement of the ROS Level

The ROS level was measured in the control, the HSV-1-treated cells, and in the same cells with the presence of EchA, EamA, and EamB using the ROS indicator DCF-DA according to prior work [8] with some changes. Treated cells were incubated with 10 μM of DCF-DA for 30 min at 37 °C in the dark. After washing twice with PBS, the intensity of DCF fluorescence of the cells was measured at an λ_ex_ of 485 nm/λ_em_ 520 nm using the PHERAstar FS plate reader (BMG Labtech, Offenburg, Germany).

For all experiments, a monolayer of Vero cells was prepared. Vero cells were seeded in 96-well plates (1 × 10^4^ cells/well) and cultured for 24 h at 37 °C in 5% CO_2_. The following experiments were performed; each was carried out in three independent replicates.

- *ROS production in HSV-1-infected cells*: Cells were treated with HSV-1 (100 PFU/mL) and cultured at 37 °C. After 1, 2, 3, and 4 h, the cells were washed with PBS and the ROS level was measured in infected (HSV-1) and uninfected cells (control).
- *ROS production in control cells with the presence of spinochromes*: The monolayer of cells treated with the tested spinochromes (5 μg/mL, 100 μL/well) and incubated for 2 h at 37 °C. After washing with PBS, the ROS level was measured. Untreated cells were used as the control.
- *ROS production in control and HSV-1-infected cells with the presence of spinochromes*: HSV-1 (100 PFU/mL) was mixed with the tested spinochromes (5 μg/mL) in a 1:1 (*v*/*v*) ratio and incubated for 1 h at 37 °C. Then, the mixture and HSV-1 (100 PFU/mL) were applied to a monolayer of Vero cells. After 1 h of incubation at 37 °C, the cells were washed with PBS and the ROS level was measured. Uninfected and HSV-1-infected cells were used as the control.

### 3.7. Molecular Docking

We used the crystal structure of echinochrome A (CCDC ID NERLUS code) [50,51]. The structures of Eam A and Eam B were obtained using the molecular editor of the MOE 2019.01 [52] program. The structures of the spinochromes were optimised with the forcefield MMFF94, and the structures of the protomers in the aqueous phase were calculated using MOE 2019.01 software. The crystal structures of the complexes of the HSV-1 gD glycoprotein with the nectin-1 receptor (PDB ID 3UKS) and the HSEA/M receptor (PDB ID 1JMA) were used as a target protein. The structure of the HS tetrasaccharide was obtained from the PDB database (PDB ID 1T8U) and used to obtain 3-O-S HS using the molecular editor MOE 2019.01. For molecular docking, a 3-O-S HS structure was used, which was solvated in the aqueous phase and optimised with the forcefield Amber10:EHT. The calculation of the electrostatic potential of the molecular surface of glycoprotein gD was carried out using the MOE 2019.01 program. Molecular docking of glycoprotein gD with the spinochromes and 3-OS HS was performed using the Dock module of the MOE 2019.01 software. The structures of 30 complexes were calculated with Score London dG, and the 10 most energetically advantageous complexes were optimised with Score GBVI/WSA dG. Contact analysis was carried out using the Ligand Interaction module of the MOE program.

### 3.8. Statistical Analysis

Statistica 10.0 software was used for statistical analysis of the experimental data. The results are given as the mean ± standard deviation (SD). Wilcoxon test was used for estimating the differences significant at *p* ≤ 0.05 between means of the control and experimental groups.

## 4. Conclusions

Thus, we found that spinochromes of the sea urchin *Echinarachnius parma* collected at a depth of more than 50 m exhibited significant anti-HSV-1 activity, mainly due to their direct virucidal properties, and their activity increased in the following order: EchA < EamA < EamB. They also inhibited the attachment of the virus to cells and, to a lesser extent, the entry of the virus into cells. One of the mechanisms of anti-HSV-1 activity of spinochromes is due to the fact that the compounds under study can directly bind to gD, competing with cellular receptors for the binding sites of this protein, thereby preventing the adsorption of HSV-1 on cells. Another mechanism of the antiviral action of the spinochromes, which have pronounced antioxidant properties, is due to a decrease in the HSV-1-induced ROS level in the cells.

Echinochrome A sodium salt is a Histochrome drug permitted for clinical application in Russia for the treatment of myocardial infarction and ophthalmological diseases. The ability of EchA to overcome the blood–brain barrier and to inactivate neurotropic viruses such as HSV-1 and tick-borne encephalitis virus makes it a prospective agent for new therapeutic use [15]. Echinamines A and B are the closest structural analogues of EchA. Their low molecular weight, comparable to EchA cytotoxicity, variety of their synthesis pathways with high yield, and more significant antiherpetic activity reported here, open perspectives to investigate their potential for clinical application.

## Figures and Tables

**Table 1:** Effects of the tested compounds at different stages of herpes simplex virus type 1 (HSV-1) infection.

Compounds	CC_50_	IC_50_ (SI)					
		Pretreatment of the Virus	Pretreatment of Cells	Attachment	Penetration	Simultaneous Treatment	Post-Infection Treatment
EchA	142 ± 6	4.1 ± 0.6 (34.6)	83 ± 14 (1.7)	33 ± 6 (4.3)	59 ± 9 (2.4)	35 ± 6 (4.1)	95 ± 18 (1.5)
EamA	146 ± 7	2.9 ± 0.4 (50.3) *	96 ± 16 (1.5)	25 ± 4 (5.8)	56 ± 8 (2.6)	34 ± 6 (4.3)	113 ± 23 (1.3)
EamB	137 ± 6	1.7 ± 0.2 (80.6) *	92 ± 14 (1.5)	16 ± 3 (8.5) *	54 ± 7 (2.5)	30 ± 5 (4.6)	80 ± 14 (1.7)
ACV	>1000	NA	NA	NA	NA	2.1 ± 0.4 (476)	0.1 ± 0.02 (10,000)

Values are presented as the means ± standard deviations. CC_50_ (μg/mL), 50% cytotoxic concentration; IC_50_ (μg/mL), concentration that inhibited 50% of HSV-1 plaque formation; SI, selectivity index (CC_50_/IC_50_); NA, no activity. * Statistically significant differences between values of EchA and the echinamines (*p* ≤ 0.05).

## Supplementary Materials

The following are available online at https://www.mdpi.com/1660-3397/18/11/550/s1, Figure S1: HPLC of products of echinochrome A amination reaction. Figure S2: The structures of echinochrome A, echinamine A and echinamine B protomers in the aqueous phase (pH 7) were calculated using the MOE 2019.01 software. Figure S3: Anti-HSV-1 activity of tested compounds in virucidal assay. Figure S4: The gD binding site, defined by the Site Finder module, that overlaps with the gD binding site for the nectin-1 cellular receptor (PDB ID 3SKU).
