# Supplementary material for: Antiviral Potential of Sea Urchin Aminated Spinochromes against Herpes Simplex Virus Type 1
Source: Mar Drugs. 2020 Nov 5;18(11):550. doi: 10.3390/md18110550 (PMC7694471; doi:10.3390/md18110550)
Supplement: Supplementary file 1 [file marinedrugs-18-00550-s001.zip › marinedrugs-982364-supplementary.docx]

***-Supporting Information–***

Antiviral Potential of Sea Urchin Aminated Spinochromes against Herpes Simplex Virus Type 1

Natalia P. Mishchenko ^1,^*^,†^, Natalia V. Krylova ^2,†^, Olga V. Iunikhina ^2^, Elena A. Vasileva ^1^,
Galina N. Likhatskaya ^1^, Evgeny A. Pislyagin ^1^, Darya V. Tarbeeva ^1^, Pavel S. Dmitrenok ^1^ and Sergey A. Fedoreyev ^1^

^1^ G.B. Elyakov Pacific Institute of Bioorganic Chemistry, Far-Eastern Branch of the Russian Academy of Sciences, 690022 Vladivostok, Russia; vasilieva_el_an@mail.ru (E.A.V.); galinlik@piboc.dvo.ru (G.N.L.); pislyagin@hotmail.com (E.A.P.); tarbeeva1988@mail.ru (D.V.T.); paveldmitrenok@mail.ru (P.S.D.); fedoreev-s@mail.ru (S.A.F.)

^2^ G.P. Somov Institute of Epidemiology and Microbiology, Far-Eastern Branch of the Russian Academy of Sciences, 690087 Vladivostok, Russia; krylovanatalya@gmail.com (N.V.K.); olga_iun@inbox.ru (O.V.I.)

***** Correspondence: mischenkonp@mail.ru; Tel.: +79-084542965

^†^ N.P.M. and N.V.K. contributed equally to this manuscript.

**TABLE OF CONTENTS:**

**The physical and spectroscropic data of spinochromes**  3

**Figure S1.** HPLC of products of echinochrome A amination reaction 4

**Figure S2**. The structures of echinochrome А, echinamine А and echinamine B tautomers in the aqueous phase 5

**Figure S3.** Anti-HSV-1 activity of tested compounds in virucidal assay 6

**Figure S4**. The gD binding site, defined by the Site Finder module, that overlaps with the gD binding site for the nectin-1 cellular receptor (PDB ID 3SKU) 7

**The physical and spectroscropic data of spinochromes**

**Echinochrome A** (7-Ethyl-2,3,5,6,8-pentahydroxy-1,4-naphthoquinone) **(EchA):** ^1^H NMR spectrum (500 MHz, CDCl_3_, δ, ppm, *J*/Hz) 1.17 (3H, t, *J* = 7.5, СН_3_), 2.73 (2Н, q, *J* = 7.5, CH_2_), 6.36 (s, ОН), 6.52 (s, ОН), 6.80 (s, ОН), 12.06 (s, ОН), 12.27 (s, ОН). ^13^C NMR spectrum (CDCl_3_, δ, ppm) 178.0, 135.8, 138.3, 180.0, 148.7, 151.3, 126.0, 161.3, 101.8, 106.3, 16.2, 12.0. The physical and spectroscropic data agree with those reported in the literature [Vasileva, E. A., Mishchenko, N. P., Vo, H. M. N., Bui, L. M., Denisenko, V. A., & Fedoreyev, S. A. (2017). Quinoid pigments from the sea urchin *Astropyga radiata*. *Chemistry of Natural Compounds*, *53*(2), 356-358.]

**Echinamine A (**3-amino-7-ethyl-2,5,6,8-tetrahydroxy-1,4-naphthoquinone) **(EamA):** C_12_H_11_NO_6_; M.m. 265. Dark brown powder; UV (EtOH) λ_max_ 217, 274, 345, 480 nm; IR (CHCl_3_) *ν*_max_ 3522, 3445, 3379 (NH_2_, β-OH), 1650, 1603 (C=O), 1589, 1562 (NH_2_, C=C) cm^-1^; ^1^H NMR spectrum (500 MHz, aceton-*d*_6_, δ, ppm, *J*/Hz) 1.14 (3H, t, *J* = 7.5, СН_3_), 2.69 (2Н, q, *J* = 7.5, CH_2_), 5.36 (2H, br. s, NH_2_), 8.49 (br. s, ОН), 9.20 (br. s, ОН), 12.62 (s, ОН), 13.02 (s, ОН). ^13^C NMR spectrum (aceton-*d*_6_, δ, ppm) 181.7, 177.4, 161.1, 154.1, 152.3, 137.0, 132.4, 126.6, 108.5, 102.8, 16.2, 12.9. HREIMS *m*/*z* 265.0598 (calcd for C_12_H_11_NO_6_, 265.0586). The physical and spectroscropic data agree with those reported in the literature [Mischenko NP, Fedoreyev SA, Pokhilo ND, Anufriev VP, Denisenko VA, Glazunov VP. (**2005**) Echinamines A and B, first aminated hydroxynaphthazarins from the sea urchin *Scaphechinus mirabilis*. *Journal of Natural Products*, ***68***, 1390–1393.].

**Echinamine B (**2-amino-7-ethyl-3,5,6,8-tetrahydroxy-1,4-naphthoquinone**) (EamB):** dark brown needles; mp 265-267 °C; UV (EtOH) λ_max_ 217, 275, 343, 480 nm; IR (CHCl_3_) *ν*_max_ 3518, 3460, 3398 (NH_2_, β-OH), 1664, 1603 (C=O), 1580, 1560 (NH_2_, C=C) cm^-1^; ^1^H NMR spectrum (500 MHz, aceton-*d*_6_, δ, ppm, J/Hz) 1.13 (3H, t, *J* = 7.5, СН_3_), 2.67 (2Н, q, *J* = 7.5, CH_2_), 5.80 (2H, br. s, NH_2_), 8.36 (br. s, ОН), 9.43 (br. s, ОН), 13.02 (2 H, s, 2ОН). ^13^C NMR spectrum (aceton-*d*_6_, δ, ppm) 176.6, 134.7, 135.1, 178.7, 154.0, 151.3, 124.4, 163.1, 103.9, 107.8, 16.6, 12.9. HREIMS *m*/*z* 265.0598 (calcd for C_12_H_11_NO_6_, 265.0586). The physical and spectroscropic data agree with those reported in the literature [Mischenko NP, Fedoreyev SA, Pokhilo ND, Anufriev VP, Denisenko VA, Glazunov VP. (**2005**) Echinamines A and B, first aminated hydroxynaphthazarins from the sea urchin *Scaphechinus mirabilis*. *Journal of Natural Products*, ***68***, 1390–1393.].


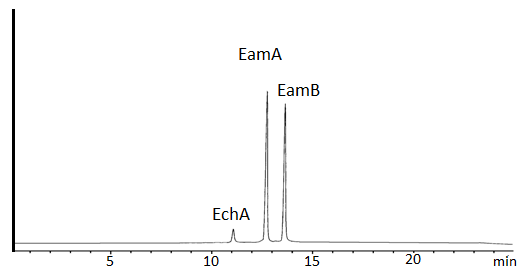


**Figure S1.** HPLC of products of echinochrome A amination reaction

| **Echinochrome А (EchA)** | | | |
| --- | --- | --- | --- |
| **32,2%** | **18,7%** | **9,4%** | **9,2%** |
| **6,7%** | **4,8%** | **4,0%** | **3,6%** |
| **Echinamine А (EamA)** | | | |
| **45.5%** | **29.8%** | **8.1%** | **2.0%** |
| **2.5%** | ****  **2.1%** |   **0%** |  |
| **Echinamine B (EamB)** | | | |
|   **77.3%** | ****  **10.0%** |  |  |
|  |  |  |  |

**Figure S2**. The structures of echinochrome А, echinamine А and echinamine B tautomers in the aqueous phase were calculated using the MOE 2019.01 software.

**Figure 3.** Anti-HSV-1 activity of tested compounds in virucidal assay. HSV-1 (100 PFU) was preincubated with various concentrations of tested compounds for 1 h at 37◦C and the mixtures were applied to the Vero cells (see details in Materials and Methods). Results are plotted as % of viral inhibition of plague formation in comparison to the untreated virus control. The values represent the mean ± standard deviations of three independent experiments. *Significant difference between values of the echinamines and echinochrome A (*p* ≤ 0.05); **significant difference between values of the echinamine A and echinamine B (*p* ≤ 0.05).


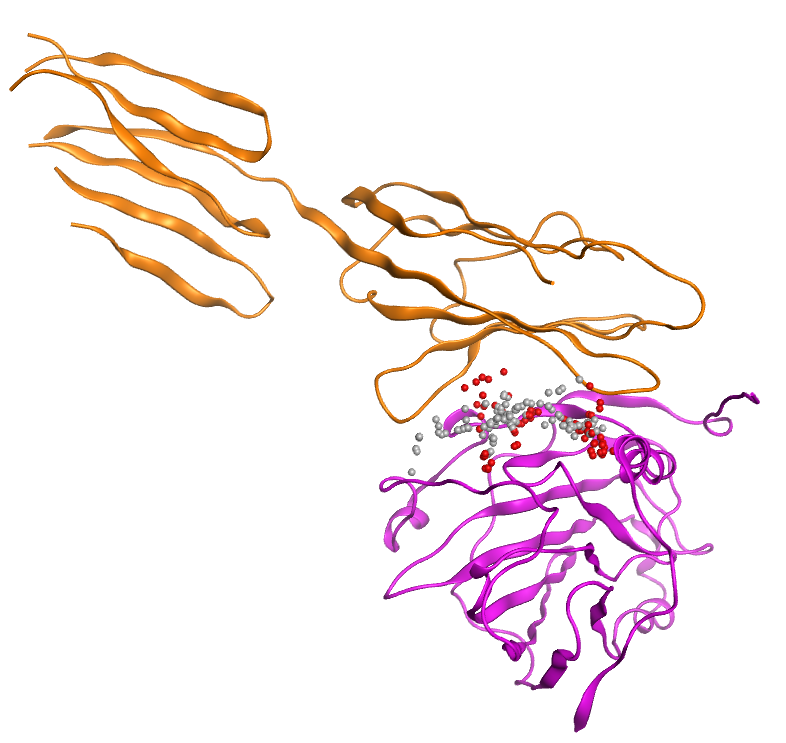


**Figure S4**. The gD binding site, defined by the Site Finder module, that overlaps with the gD binding site for the nectin-1 cellular receptor (PDB ID 3SKU). Protein structures are shown in ribbon form, the gD molecule is shown in pink and the nectin-1 receptor fragment is shown in orange.
